# Water Treatment for Fish Aquaculture System by Biochar-Supplemented Planting Panel System

**DOI:** 10.1155/2020/7901362

**Published:** 2020-08-28

**Authors:** Sumrit Mopoung, Vijitr Udeye, Supaluck Viruhpintu, Nonglak Yimtragool, Visarut Unhong

**Affiliations:** ^1^Department of Chemistry, Faculty of Science, Naresuan University, Phitsanulok, Thailand; ^2^Department of Biology, Faculty of Science, Naresuan University, Phitsanulok, Thailand

## Abstract

Rice husk biochars were prepared by carbonization at 400–600°C. The products were analyzed by FTIR, SEM-EDS, BET, and approximate analysis in order to find final products with the best properties and the lowest carbonization temperature. It has been found that the biochar prepared at 500°C, which has 37.86 ± 0.11% yield, 341.0776 m^2^/g of BET surface area, and 0.136639 cm^3^/g of micropore volume, is suitable for use as a root supplement in the aquaponic system. The aquaponic systems consist of aquaculture and a hydroponic system with and without biochar supplement. The control experiment consists of an aquaculture and planting panel with biochar supplement disconnected from each other. Tilapia and Chinese morning glory were used for growth studies. The water quality from all aquaculture ponds has also been analyzed at an interval of 10 days for 47 days. The results showed that the growth rates of Tilapia and Chinese morning glory in the aquaponic system with biochar were clearly higher than in the control experiment, which is in accordance with the water quality in each aquaculture pond. However, the growth rates of Tilapia (23.5 g/body vs. 22.7 g/body) and morning glory (3.907 g/stem vs. 2.609 g/stem) in supplemented biochar system tend to be higher than the nonsupplemented biochar system. It has been shown that rice husk biochar can help in treating water in the aquaponic system by increasing the amount of dissolved oxygen in the aquaculture water and conversion of toxic compounds to those beneficial for plant growth.

## 1. Introduction

Biochar is a carbon-rich material which is produced from the pyrolysis process of biomass in a closed system using the reduction reaction [1]. Biochar materials have high adsorption for water and nutrients and are highly stable [2]. Biochar materials could increase plant growth by improving the physicochemical and biological properties of soil and retained soil fertility. Furthermore, biochar materials can also remediate organic/inorganic contaminants. Biochar has been used as hydroponic substrate for production of leafy vegetables [1]. It has also been added to sand for improved water and fertilizer retention for plant growth [2]. At present, aquaculture is utilized for fish production and has been very popular in the past decades. It has been developed to increase productivity by increasing the density of fish, which has caused problem of wastewater release to ecology systems [3, 4]. Therefore, it is of interest to develop solutions, which will reduce wastewater problems from aquaculture systems. One solution to this problem is to connect an aquaculture system to a hydroponics system [5], in which hydroponic plants could absorb and uptake soluble compounds from the wastewater [6]. The soluble compounds have two major components. These are nitrogen and phosphorus compounds, which originate from fish feed. The nitrogen and phosphorus are retained in the body of the fish and some quantities are transferred into the culture water [4]. The combination of aquaculture (fish production) and hydroponics (plants production) is called aquaponics, which is beneficial as it reduces the use of resources (water, nutrients, and land) and also reduces the operation area and wastewater discharge to the environment. In addition, the products from aquaponics are organic fruits and vegetables [7, 8]. In an aquaponic system, the aquaculture operation could be benefited by improving the quality of water, which increases stocking densities, or by reducing costs associated with treating the effluent. On the other hand, the hydroponic operation can benefit from the reduction of fertilizer inputs and labor or facilities needed to maintain adequate moisture levels [5]. However, wastewater improvement and water reuse ability in aquaponics vary depending on the hydroponic medium, hydraulic loading rate, plant species, and the plant: fish ratio [9]. One of the problems that occurs in the hydroponic part is the limited ability to remove suspended solids, which is due to the lack of culture media that can trap solids [6]. Growth media for hydroponic system could remove the suspended solids and could as well create a nutrient pool and provide adequate air space for respiration around the plant roots [10]. Cork, rice hulls, peat, coir fiber [11], Palm kernel shells, and periwinkle shells [10] have been used as growth media for hydroponic systems. These growth media are still unstable and less porous than charcoal.

Thus, this research will study the use of rice husk biochar, which is stable and can be produced using simple procedure in high quantity. The rice husk biochar was used as growth medium for hydroponic system in aquaponics. The hydroponic system with rice husk biochar was studied in comparison to hydroponic system without rice husk biochar and only with rice husk biochar for planting. The growth rates of fish and plants and the quality of water in aquaponics were studied.

## 2. Materials and Methods

### 2.1. Preparation and Analyzation of Biochar from Rice Husk

The rice husk was carbonized at 400, 500, and 600°C for 1 h. The rice husk biochar products and rice husk were characterized by Fourier Transform Infrared Spectrometer (FTIR, Spectrum GX, Perkin Elmer, USA), Scanning Electron Microscope coupled with Energy Dispersive X-Ray Spectrometer (SEM-EDS, a LEO 1455 VP Electron Microscopy, England), and surface area and porosity analyzer using BET gas adsorption (Micromeritics TriStar II). The approximate analysis of samples was also used for their analysis. The biochar product with the best characteristics and lowest carbonization temperature was collected as growth material for planting panel system.

### 2.2. The Aquaponic Construction

**Figure 1:**
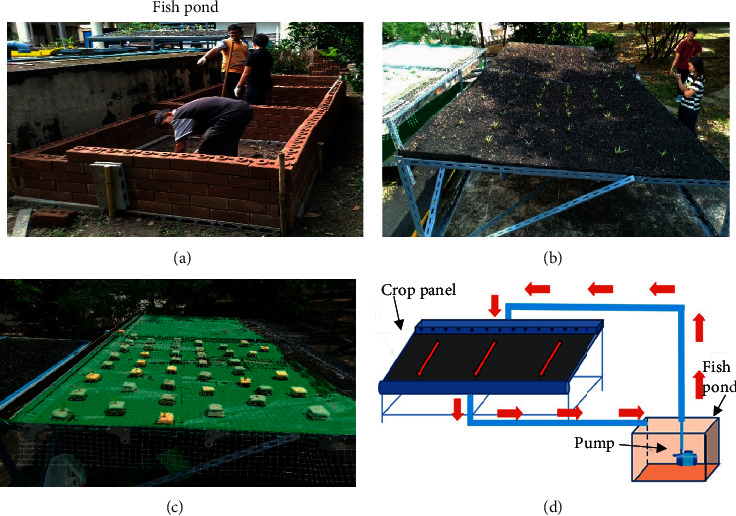
(a) Aquatic pond, (b) the first crop panel, (c) the second crop panel, and (d) the third crop panel.

Three aquatic ponds with width, length, and height of 2 m, 2 m, and 70 cm, respectively were constructed using interlocking bricks (10 cm × 10 cm × 20 cm) (Figure 1(a)). A plastic sheet was used to cover the bottom and sides, up to 50 cm depth, of the brick structure to enable water storage (volume 2 m^3^) inside each pond. Subsequently, three crop panels were also constructed with width and length of 2 m and 2 m, respectively, and a 2% slope. Each panel is 30 cm above the aquatic pond and covered with plastic. The first crop panel (Figure 1(b)) was filled with 15 cm of rice husk biochar and was not connected to an aquatic pond. This panel represents the control experiment. The first aquatic pond also acted as a control experiment. The second crop panel was covered with a net, equipped with planting sponges placed 15 cm apart (Figure 1(c)), and connected to the second aquatic pond. Finally, the third crop panel was filled with 15 cm of rice husk biochar and connected to an aquatic pond (Figure 1(d)). Morning glory (*Ipomoea aquatica*) was planted in each crop panel with individual plant separation of 15 cm × 15 cm. For aquatic system, 200 climbing perch fish (*Anabas testudineus*) were raised in each pond and fed twice a day (morning-evening) with 40% protein in a food mixture which amounted to 3% total weight of the fish. The water in the first and second pond was circulated with a rate flow of 200 L/h using a pump and flowing through the second and third crop panels, respectively. The first pond was constructed in the same way as the second and third ponds but without a connection to a crop panel. The growth rates of morning glory (height and number of leaves) and climbing perch (body weight and body length) for all experiments were measured every week during the 47 days of the experiment. At the same time, the pH (Mettler Toledo), total solids [12], suspended solids [12], nitrite by spectrophotometer (double beam, Jusco V650) at 540 nm, phosphorus in the orthophosphate formed by spectrophotometer at 880 nm, and dissolved oxygen (DO) by titration with sodium thiosulphate [13] of water from all aquatic ponds were also analyzed at an interval of 10 days.

## 3. Results and Discussion

The percent yield of rice husk biochar decreased with increasing carbonization temperature from 400°C to 600°C (Table 1). However, the percent yield of biochar remained steady when the carbonization temperature was increased from 500°C to 600°C. This is because the hemicellulose, cellulose, and lignin decompose at temperatures below 500°C [14]. This is associated with the decrease of volatile matter observed upon increasing the carbonization temperature from 400^o^C to 500°C. The volatile matter content then remains nearly constant upon the increase of carbonization temperature to 600°C. Therefore, it can be said that the temperature needed to achieve complete carbonization of rice husk is 500°C, which gives a yield of 37.86 ± 0.11%. The high content of fixed carbon and low content of volatile matter (<10%) of rice husk biochar prepared using carbonization at 500°C indicate that the rice husk biochar is stable and suitable for use as adsorbent in the environment [14]. So, the rice husk biochar prepared at 500°C was collected as supplemented material for the hydroponic system.

### 3.1. Result of FT-IR Analysis

**Figure 2:**
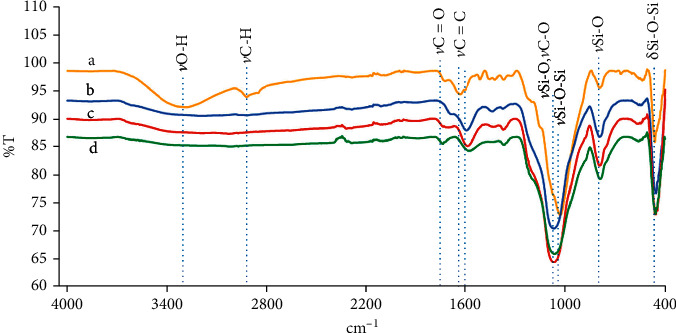
FTIR transmission spectra of (a) rice husk, (b) rice husk biochar at 400°C, (c) rice husk biochar at 500°C, and (d) rice husk biochar at 600°C.

The FTIR transmission spectrum of rice husk (Figure 2) shows bands at 3239 cm^−1^, 2919 cm^−1^, 1745 cm^−1^, and 1660 cm^−1^ corresponding to *ν* O-H of an acid or alcohol, the aliphatic and hydroaromatic residual C-H alkyl chains of cellulose, hemicellulose and lignin, carbonyl groups in the stretching mode of aldehydes and ketones in the association of hemicelluloses with lignin, and carbonyl (*ν*-C=O) of the esters in hemicellulose, and C=C bonds in organic components [15], respectively. The bands at 1745 cm^−1^ and 1660 cm^−1^ are also related to the elastic vibrations of the CO bond in carboxyl groups [16]. The spectrum also contains other bands at 1040 cm^−1^, 806 cm^−1^, and 465 cm^−1^, which correspond to the biogenic silica [14, 17–19]. The band at 1040 cm^−1^ is associated with the asymmetric stretching vibration of Si-O-Si in the biogenic silica. This band also corresponds to *ν*CO and *δ*CO [16]. Finally, the bands at 806 cm^−1^ and 465 cm^−1^ correspond to the symmetric vibrations of the Si-O bonds in the silicon-oxygen tetrahedrons (SiO_4_) and Si-O-Si bending vibrations [15]. After the carbonization process at 400–600°C, the FTIR bands of the rice husk biochar obtained at 400°C (Figure 2) show a band at 1090 cm^−1^, which shifted from 1040 cm^−1^ and corresponds to the biogenic silica. It is a characteristic of the tridymite, which is due to the *ν* Si-O. The peaks at 804 cm^−1^ and 465 cm^−1^ related to the ring structure of the SiO_4_ tetrahedra of silica belonging to the cristobalite type and *δ* Si-O-Si, respectively [16]. Furthermore, it was observed that the bands of *ν*O-H, *ν*C-H, and *ν*C=O of hemicellulose, cellulose, and lignin disappeared, while the bands of *ν* C=O (1710 cm^−1^) and *ν*C=C (1615 cm^−1^) began to appear. This shows that organic substances have decomposed and that aromatic carbon structures begin to develop at 400°C. Reactions leading to the development of aromatic structures during carbonization are dehydration, demethylation, demethoxylation, decarbonylation, and decarboxylation of hydroxyl groups and other oxygen-containing functional groups. The results of these reactions cause the development of C=C bonds and smaller aromatic rings [14], which is confirmed by the increased intensity of the band at 1615 cm-1. Similarly, the rice husk biochars prepared at 500–600°C (Figure 2) show more characteristics of biochar and silica with dominant *ν*C=O, *ν* C=C, *ν*Si-O, and *δ*Si-O-Si bands. The bands of *ν*C=O, *ν*C=C, especially, become more separated changing their positions from 1618 cm^−1^ to 1708 cm^−1^ and 1615 cm^−1^ to 1755 cm^−1^. This indicates the formation of more stable condensed carbon structures.

### 3.2. Result of SEM-EDS Analysis

**Figure 3:**
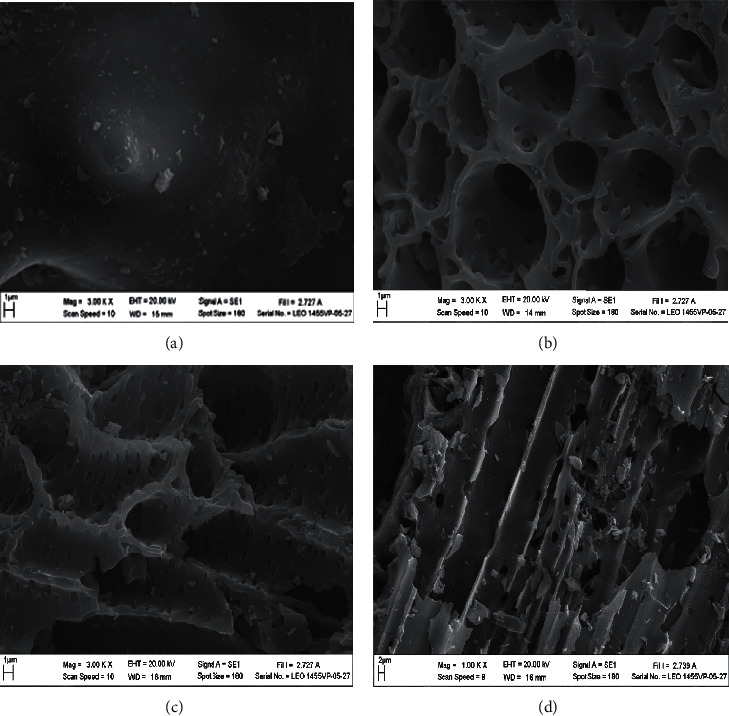
SEM images of (a) rice husk biochar prepared at 400°C, (b) rice husk biochar prepared at 500°C, (c) rice husk biochar prepared at 600°C, and (d) rice husk biochar prepared at 500°C after being used as supplemented plant root for 47 days.

Figure 3 shows SEM images of rice husk biochar prepared by carbonization at temperatures between 400°C and 600°C. The image showed that rice husk biochar prepared at 400°C (Figure 3(a)) has smooth embossed surface with some small particles. However, increasing the carbonization temperature to 500°C and 600°C results in the formation of hollow tunnels with open pores. The presence of small particles on the walls of the tunnels of the biochar can be observed. These results are related to the degradation of volatile matter, for example, cellulose, hemicellulose, and lignin. For example, hemicellulose decomposes between 220°C and 350°C, cellulose decomposes between 315°C and 400°C, and lignin decomposes above 400°C [14]. It has been reported that the completion of charcoal formation is due to the complete pyrolysis of lignin [16]. Therefore, the open pores are observed in rice husk biochars prepared at temperatures above 400°C but did not appear on the surface of rice husk biochar prepared at 400°C. These results correspond to the results of FTIR in which the functional groups belonging to cellulose, hemicellulose, and lignin of biochar disappear for materials prepared at 500°C and 600°C. As can be seen from the results of EDS (Table 2), the carbon content of rice husk biochar materials prepared at 500–600°C is constant with low oxygen content. This confirms that the degree of carbonization of rice husk is completed at about 500°C. In addition, the amount of Si of biochar is quite high which is consistent with the results of FTIR.

### 3.3. Surface Area and Porosity Analysis of Rice Husk Biochar

Data on surface area and porosity of rice husk biochar are shown in Table 3. It can be seen that the surface area and pore volume of biochar prepared at 400°C are very low. This confirms that the carbonization of rice husk at 400°C results in incomplete biochar production which corresponds to the results of the above analyses especially images from SEM (Figure 3(a)). For biochars prepared by carbonization of rice husk at 500–600°C, the surface area and pore volume (micropore and mesopore) are drastically increased. The content of mesopores, especially, increases upon increasing the carbonization temperature from 500°C to 600°C. This is caused by complete degradation of volatile matter above 400°C. Furthermore, the surface area and volume of mesopores are highly increased between 500°C and 600°C. This is the result of secondary decomposition of the biochar components together with the collapse of the microporous structures, which consequently pave the way for the formation of the mesoporous structure when the pyrolysis temperature is increased [14]. However, surface area and pore volume of rice husk biochars prepared at 500–600°C are still low. This may be caused by the fact that the pores of the biochar contain fine particles and ash as shown in the SEM image (Figures 3(b) and 3(c)).

### 3.4. Water Analysis

Table 4 shows the data of water analysis resulting from aquatic ponds numbers 1–3 on 7^th^–47^th^ days. This shows that the supplementation of biochar in the plant panel connected to the aquatic pond (number 3) could reduce the total solid, suspended solid, nitrite, and orthophosphate content in water samples as compared to the aquatic pond (number 1) and aquatic pond connect to hydroponic system without rice husk biochar (number 2) while it could increase the dissolved oxygen (DO) content in the water samples. This is because the suspended solids, which were the cause of water turbidity [8], were filtered by biochar and plant roots while dissolved solid, nitrite, and orthophosphate were adsorbed by the rice husk biochar and absorbed by plant root for growth. However, this content is more reduced after 17^th^ day. This is attributed to filtering and absorption by root of the Chinese morning glory after full growth. The suspended solid was more extensively filtered as there are more roots, which have spread all over the biochar, while some of the dissolved solids, nitrite, and orthophosphate were used for the growth of the root, stem, and leaf of Chinese morning glory as well as bacteria [20]. This phenomenon has occurred in the aquatic pond connected to the hydroponic system without rice husk biochar (number 2) as well. It can be seen that the nitrite content in water of the aquatic pond connect to plant panel with rice husk biochar has a value not exceeding the specified (2.0 mg/L) for fish pond water [20]. This shows that supplementing biochar has an important role in the elimination of these substances in aquatic pond. However, the hydroponic system without biochar can also reduce the nitrite amount below 2.0 mg/L for a substantial amount of time. This is because, during the long planting time, the Chinese morning glory root becomes enlarged, which can increase the surface area for absorption of nitrite (in nitrate form that is converted from nitrite by nitrification). The content of orthophosphate has increased steadily with experimental duration for all aquaponic ponds. This is because plants use high amount of phosphate for root growth at the early stage of development [20]. The dissolved oxygen value exhibits an increasing trend from pond 1 to pond 3. There were water circulating systems, which can increase contact of water and atmosphere air, installed in all ponds. The addition of biochar in the plant bed system, especially, can increase the contact surface area between water and air. This is caused by high porosity and surface area of the biochar that can be a channel for air and create contact surfaces. In all cases, the DO levels are higher than the lowest levels (5 mg/L) required for warm water fish farming [8]. However, the low DO in aquatic pond number 1 is due to a higher microorganism activity and higher decomposition of soluble solids, which use high amounts of oxygen [21]. Finally, the pH values of the aquatic waters in all ponds are in the very weakly acidic to neutral range. The value increases slightly from pond 1 to pond 3. It shows that acidic substances made by fish farming (CO_2_ from fish breathing) have been eliminated by absorption in the plants and the adsorption on the biochar. However, the pH values of the water from all fish ponds are still within the limited range (6.5–9.0) which is an optimum condition for the conversion of ammonia to nitrite by bacteria. It is also in the normal range for the hydroponic system, which is generally between 5 and 7.5 [20].

## 4. Growth of Plant and Fish

### 4.1. Growth of Plant

Table 5 shows data for the growth of the Chinese morning glory after planting for 47 days (normally, it takes only 21 days to grow until harvesting). It can be seen that all of the growth parameters of the Chinese morning glory for crop panel number 3 (supplemented with rice husk biochar and connected to aquatic pond) are higher than crop panel number 1 (plant bed supplemented with rice husk biochar and disconnect from aquatic pond) and crop panel number 2 (without biochar and connected to aquatic pond). The growth of the Chinese morning glory in crop panel number 1 is low. This is because crop panel number 1, which is a control experiment, was not supplemented with a fertilizer and did not have water from the circulating aquatic system. However, the growth parameters of both crop panels numbers 2 and 3 are almost equal. This shows that the Chinese morning glory of both crop panels received roughly equal number of nutrients for growth from the circulating water. It has been shown that high plant growth results in a large number of substances in the water being used. Long plant roots, especially, which have a high surface area could absorb nutrients with high efficiency. As reported by Estim et al. [20], plants with larger root surface area provide greater surface area for attachment of microbial organisms that convert ammonia to nitrite and nitrite to nitrate. In addition, high concentration of DO is one of the factors that could be leading to increased mass of the plants. It can be seen that the amount of nitrite in the aquatic ponds 2 and 3 is low in comparison to aquatic pond 1, while the DO is high. Moreover, using rice husk biochar as a root supplement increased the efficiency of water treatment and nutrient absorption of plant roots by providing surface area for attachment of bacteria. Furthermore, considering the plant root growth, it can be seen that the plant roots in aquatic pond 2 exhibit higher growth than the plant roots in aquatic pond 3. This may be due to the use of sponge as a root supplement for plant panel 2, which is not distributed completely throughout planting panels, causing limited amount of water around the roots. On the other hand, the plant panel connected to aquatic pond 3 used rice husk biochar to spread water evenly in the full area of the plant panel. In this panel, water and nutrient absorption proceed efficiently, which facilitates the beneficial bacterial activity [20]. Therefore, the plant roots in the plant panel 2 were more dispersed than in the plant panel 3. The growth of the stems and leaves in the planting panels 2 and 3 is high due to the high growth of plant fine roots [22].

### 4.2. Growth of Fish

**Figure 4:**
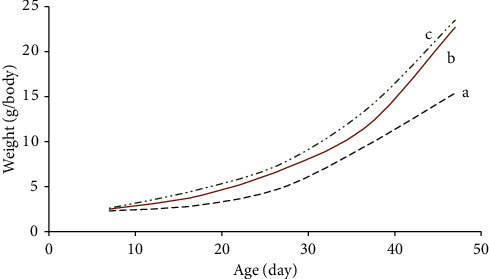
Graph showing the growth of fish by the time in (a) pond number 1, (b) pond number 2, and (c) pond number 3.

Figure 4 shows the graph of fish growth over the 47 days. The graph shows that the growth of fish in pond number 1 (Figure 4) is slower than in ponds numbers 2 and 3 (Figure 4) throughout the trial period. This difference between pond 1 and ponds 2 and 3 becomes more evident with increasing farming time. The growth of fish in ponds numbers 2 and 3 are slightly different and tend to come closer together with extended periods of time. This is because of the good quality of water in aquatic culture pond numbers 2 and 3, which is caused by plants absorbing compounds that are toxic to fish from aquatic water. This can be seen from the good growth of the plants. Therefore, the resulting water quality is better for the fish growth in aquatic ponds 2 and 3 in comparison to aquatic pond 1. For aquatic pond 1, water is not passed through the planting panel and therefore substances which can be toxic to fish are not being eliminated in this case. These effects cause the fish to have higher stress. For aquatic ponds 2 and 3, fish in both aquatic ponds grow with similar rate, especially at longer time points. This is because the plant roots have spread more extensively in the planting panel 2 at later times. However, the growth of fish in aquatic pond 3 is still likely better than aquatic pond 2. This shows that rice husk biochar has effect on water treatment as it provides surface area for attachment of bacteria that help in conversion of toxic chemicals into nontoxic substances, improving water quality and absorption of nutrients through the plant roots [20].

### 4.3. SEM and EDS of Biochar after Being Used for Water Treatment

Figure 3(d) and Table 2 show the SEM image and elemental composition of rice husk biochar prepared at 500°C after its use as supplemental plant root material for 47 days. The results indicate that some mass of biochar has been destroyed, which may be caused by insertion of the plant fibrous roots and swelling with water. As a result, decreased amount of C and increased amount of Si are observed. In addition, the amount of O is also increased in the used material, which may be related to the increase in DO values in aquatic water and oxidation of plant roots. The contents of P and Ca are derived from the feed digestion by fish, which may get accumulated on the biochar.

## 5. Conclusion

Investigation of the preparation of biochar at carbonization temperatures in the range 400–600°C found that the biochar prepared at 500°C had stable and porous properties making it suitable to act as root supplement in the planting system. The biochar material was prepared with the yield of 37.86 ± 0.11%. Using this biochar as a root supplement in the aquaponic system indicated that the growth of Tilapia and Chinese morning glory was increased in its presence in comparison to experiments without the inclusion of the biochar. Clear differences are observed especially with the control systems of isolated aquaculture and planting panel, where the growth of both Tilapia and morning glory was clearly retarded. Rice husk biochar has improved the water quality in the aquaculture system by helping to maintain the DO level above the specified limit of 6.40–7.40 mg/L while the nitrite content remained below the toxicity level of 0.1216–0.1641 mg/L. The biochar, also a tendency, reduces the number of total solids and suspended solids in the water. The biochar can act as a filter and adsorbent due to its porosity and high surface area. Rice husk biochar also helped plant roots get more nutrients for root and stem growth as the biochar is able to store nutrients well. In this regard, as the time frame for fish farming and plant growth in the aquaponic system increases, the efficiency of water treatment in the system is improved as the roots of plants grow and spread out more.

## Figures and Tables

**Table 1:** Proximate analysis and % yield of rice husk and rice husk biochar products.

Sample	Proximate analysis				% yield
	% moisture content	% volatile matter	% fixed carbon	% ash	
Rice husk	10.08 ± 0.62	69.98 ± 2.85	9.85 ± 0.68	10.08 ± 0.27	—
Rice husk biochar prepared at 400°C	2.83 ± 0.31	20.13 ± 1.32	56.75 ± 0.54	20.29 ± 0.43	43.23 ± 0.10
Rice husk biochar prepared at 500°C	2.81 ± 0.57	7.36 ± 0.72	68.64 ± 2.32	21.19 ± 0.36	37.86 ± 0.11
Rice husk biochar prepared at 600°C	2.61 ± 0.52	5.85 ± 0.75	69.46 ± 2.74	22.08 ± 0.31	37.41 ± 0.38

**Table 2:** Elemental composition of rice husk biochar from EDS.

Samples of biochar prepared at	Elements composition (%wt)					
	C	O	Si	K	P	Ca
400°C	74.62	22.02	2.94	0.42	—	—
500°C	77.44	16.54	5.39	0.63	—	—
600°C	78.41	13.71	6.95	0.92	—	—
After use	62.20	18.90	16.92	—	1.11	0.86

**Table 3:** Surface area and porosity of rice husk biochar by BET.

Samples of biochar prepared at	BET surface area (m^2^/g)	Micropore volume (cm^3^/g)	Surface area of pores between 17 Å and 3,000 Å (m^2^/g)	volume of pores between 17 Å and 3,000 Å (cm^3^/g)
400°C	7.6311	0.000278	2.2491	0.006805
500°C	341.0776	0.136639	23.0061	0.024664
600°C	414.5242	0.149359	31.7760	0.025080

**Table 4:** Total solids, suspended solids, nitrite, orthophosphate, dissolved oxygen, and pH of water in aquatic ponds and hydroponic system.

Pond number	Total solids (mg/L)	Suspended solids (mg/L)	Nitrite (mg/L)	Orthophosphate (mg/L)	Dissolved oxygen (mg/L)	pH
7^th^ day						
1	162	94	1.2083	0.2347	5.34	6.542
2	150	80	0.9439	0.2234	6.35	6.568
3	132	54	0.1613	0.1435	6.41	6.983

17^th^ day						
1	204	172	2.1611	0.3253	5.73	6.573
2	83	96	1.1123	0.2023	6.65	6.751
3	56	43	0.1216	0.1364	7.34	7.163

27^th^ day						
1	290	228	3.6583	0.3425	5.54	6.625
2	72	64	2.8732	0.1958	6.52	6.356
3	42	34	0.1486	0.1743	7.31	6.923

37^th^ day						
1	328	280	3.0708	0.4173	5.38	6.831
2	60	47	1.6521	0.2042	6.75	6.259
3	36	25	0.1209	0.1725	6.40	7.038

47^th^ day						
1	373	302	3.1937	0.4264	5.30	6.878
2	43	32	1.6190	0.2273	6.78	7.078
3	35	28	0.1641	0.1755	7.40	7.048

Pond number 1 = aquatic pond, pond number 2 = aquatic pond connect to hydroponic system without rice husk biochar, and pond number 3 = aquatic pond connect to planting panel with rice husk biochar.

**Table 5:** The results of the growth measurement of the morning glory after 47 days.

Crop panel number	Stem height (cm)	Number of leaves (stem)	Fresh stem weight (g/stem)	Fresh root weight (g/stem)	Dried stem weight (g/stem)	Dried root weight (g/stem)	Trunk diameter (cm)
1	16.34	6.6	1.000	0.542	0.1402	0.0560	0.25
2	23.65	8.9	2.609	1.899	0.3710	0.1390	0.48
3	23.77	9.7	3.907	1.418	0.3872	0.1130	0.47

## Data Availability

The data used to support the findings of this study are included within the article.
